# Perspectives Regarding the Privacy, Security, and Confidentiality of Data Collected via mHealth Apps in Saudi Arabia: Qualitative Analysis

**DOI:** 10.2196/83381

**Published:** 2026-04-23

**Authors:** Nasser Alhammad, Mohannad Alajlani, Alaa Abd-alrazaq, Theodoros Arvanitis, Gregory Epiphaniou

**Affiliations:** 1 Department of Health Informatics, Saudi Electronic University, Jeddah, Saudi Arabia; 2 Institute of Digital Healthcare, Warwick Manufacturing Group (WMG), University of Warwick, Millburn House, Coventry, CV74AL, United Kingdom, 44 558885007, 44 7563338064; 3 Warwick Manufacturing Group (WMG), University of Warwick, Coventry, United Kingdom; 4 AI Center for Precision Health, Weill Cornell Medicine, Doha, Qatar; 5 School of Engineering, University of Birmingham, Birmingham, United Kingdom

**Keywords:** awareness, data privacy, confidentiality, security, health care, patients, mHealth, mobile apps, mobile phone

## Abstract

**Background:**

As Saudi Arabia aims to enhance the adoption and utilization of mobile health (mHealth) apps in the health care sector, it is vital to identify the potential challenges that may be faced in the process and how to address them.

**Objective:**

This study aimed to explore patients’ and stakeholders’ perspectives regarding the privacy, security, and confidentiality of data collected via mHealth apps in Saudi Arabia.

**Methods:**

A qualitative research design was used by conducting an in-depth interview with 25 participants, comprising health care workers (HCWs), patients, and mHealth app developers. The interview questions focused on awareness of mHealth apps and issues relating to data security, privacy and confidentiality, benefits of mHealth apps, challenges faced, and features that may improve the utilization of such digital health technologies. Thematic analysis was performed using NVivo software (version 12; QSR International).

**Results:**

Patients were relatively less informed regarding events associated with data privacy and security than HCWs. Specific factors were identified to influence patient awareness with HCWs, suggesting the need for patient education and collaboration with cybersecurity organizations. Participants posited that advanced security features, user-friendly features, online consultation for emergencies, remote monitoring features, and taking patient needs into account are important facilitators of mHealth apps. Shared experiences mainly revolved around the accessibility to information and reliability of mHealth apps, while the challenges were related to poor usability, technical difficulties, data security, and data breaches. Patient autonomy, remote monitoring, self-care management, medical adherence, and time and cost-saving summed up the perceived benefits of the mHealth app.

**Conclusions:**

These findings may assist policymakers in developing strategies to improve Saudi users’ and patients’ adoption of mHealth apps and address the concerns raised to benefit significantly from these advanced health care modalities.

## Introduction

With the introduction of smartphones and efforts to improve the delivery of health care services, mobile health (mHealth) apps have witnessed a wider acceptance by the public and health care workers (HCWs) [1,2]. The outbreak of COVID-19 also catalyzed the dependence on digital health as mHealth apps were used in diverse aspects of health care delivery, such as disease management, self-monitoring, health information gathering, supervision of behavioral changes, fitness management, and reminding patients of rehabilitation and medication schedules [3,4]. mHealth apps are also helpful in managing health data, providing easy access to health records, and offering opportunities to execute mobile consultation and remote monitoring either during treatment or follow-up assessment [5].

HCWs and patients constitute the predominant stakeholders within the health care sector. mHealth apps facilitate the sharing of clinical data and information between these 2 bodies [6]. Such clinical data are available in the clinical information systems (CISs), which are specific domains in the hospital information systems, directly linked to inpatient and outpatient care. These systems are usually connected to other subsystems within the health care facility, such as department units and laboratories [7]. In situations where CISs are the main sources of decision-making and patient management for clinicians, both the accuracy of information provided by patients and how such data are stored and secured are pertinent. Despite the significant roles of integrating mHealth apps and CISs, particularly in terms of hospital workflows, issues relating to data security, confidentiality, and privacy remain a significant challenge. A few studies have demonstrated the adverse effects of data confidentiality, privacy, security, and regulatory supervision of mHealth apps on the adoption and implementation of these digital health technologies [8].

Patients’ and HCWs’ awareness about data confidentiality and privacy may influence the risk of data breaches. Moreover, these end users have an obligation for the privacy and security of their data to be guaranteed [4]. This data-related relationship between HCWs and patients is contractual and should comply with data privacy regulations, such as the Health Insurance Portability and Accountability Act (HIPAA) and the General Data Protection Regulation (GDPR). Developers of mHealth apps are also encouraged to implement secure authentication and data encryption and carry out timely risk assessments to ensure data security and privacy [9,10]. The HIPAA entails the administrative, physical, and technical aspects to facilitate the privacy of security of personal health data, whereas the GDPR emphasizes the documentation of users’ consent before recording their personal data and preserving their right to access, amend, delete, or restrict processing their data [10,11]. While mHealth providers are more likely to be aware of the security measures, privacy, and confidentiality related to patients’ health data, patients and end users appear to be lagging in these important aspects [4,10]. Users’ knowledge and awareness of these aforementioned provisions may influence their perspectives and adoption of mHealth apps.

In Saudi Arabia’s context, the increasing population of smartphone users has facilitated the implementation of several mHealth apps aligning with the country’s Vision 2030 goals [11,12]. This transformation to digital health management commenced in 2017 to enhance the delivery of health care services in public and private health [13,14]. However, the adoption of mHealth apps in Saudi Arabia’s health care sector is yet to meet the expected benchmark, with issues relating to data privacy and security as one of the mitigating factors [11,15]. While this problem is evident from recent studies using qualitative research methods [11,15,16], the underlying reasons are not fully understood, particularly in the context of Saudi Arabia [16].

Another issue regarding existing literature on mHealth apps in Saudi Arabia is that most previous studies have focused more on health care professionals, whereas patients’ perspectives have received little attention [15]. Both developers’ and users’ perspectives need to be balanced to ensure that mHealth apps achieve their targeted goals in enhancing health care service delivery. Qualitative approaches, such as in-depth interviews, focus group discussions, and interpretative phenomenological techniques, offer diverse opportunities to elucidate the underlying events contributing to data privacy and security issues. Such information is pertinent in addressing users’ needs and developing patient-friendly mHealth apps in Saudi Arabia. This study aims to explore patients’ and stakeholders’ perspectives regarding the privacy, confidentiality, and security of data collected via mHealth apps in Saudi Arabia.

## Methods

### Study Design and Location

To achieve the aim of exploring the use and perceptions of mHealth applications among health care workers and patients, a qualitative study was conducted. Specifically, interviews were carried out to gather in-depth information from HCWs and patients using mHealth apps. The research took place in regions where hospitals feature cutting-edge facilities and currently use mHealth apps for patient management (Riyadh, Madinah, Mecca, Tabuk, Najran, Hall, Northern, Eastern, Al Jouf, Asir, Qassim, Jazan, and Al Baha). These apps are mainly categorized into 4 types: government apps, hospital, insurance, and private clinic apps [14]. Examples of these medical applications include Sehhaty, Qariboon, Health Volunteering, SFHP Riyadh, Tawuniya, Labayh, Al Mostajeeb, and Meena Health, which were introduced in the last 6 years. The Sehhaty app, also known as “My health,” is a robust digital health platform that offers personal health information and promotes knowledge about healthy lifestyles and public health. It was later used for booking COVID-19 vaccine appointments [17,18]. In terms of core functionalities, most of the mHealth apps are designed for general health, such as support or wellness, care delivery, and telemedicine services. Meanwhile, platforms such as Labayh emphasize health education and mental health consultations, addressing specific areas in the health care service delivery [14,17].

### Study Population

The study population and unit of analysis in this study encompass all the HCWs, users and nonusers, and developers of mHealth apps who fulfill the inclusion criteria.

### Participant Selection

The study used a nonprobability sampling method, specifically purposive sampling, to select participants. Eligible participants included HCWs from hospitals offering mHealth apps for patient management in the identified provinces, patients who are either currently using these apps or nonusers, and developers of these apps. We decided to recruit nonusers because they can equally provide important insights, particularly regarding the barriers to adoption of mHealth apps. mHealth app users are generally classified as either active or passive according to their present usage status. Active users are highly engaged and use the complete provisions in mHealth apps, whereas passive users use the apps occasionally and restrict themselves to a few features [19]. Additionally, participants needed to be aged 18 years or older and willing to give both oral and written consent to take part in the research. For qualitative research, sample sizes are not definite but are still required in selecting the study participants [20]. According to Leedy and Omrod [20], the sample size in qualitative research should range from 5 to 30. Therefore, our target was to recruit about 20‐30 respondents, depending on the attainment of data saturation. Multidimensional approaches, including emails and social media posts, were used to identify and invite participants in this study.

### Data Collection Method

To ensure systematic and organized interviews, an interview guide was developed by the principal investigator (NA) and the supervisor team. Specifically, questions in the interview session were synthesized based on an in-depth review of previous literature and discussion between the researchers. Various studies using either interviews or focus group discussions were reviewed before selecting and modifying the topics, aligning with this study context [4,21-24].

Overall, 2 interview guide sections were developed: 1 for the users and nonusers of mHealth apps and 1 for the HCWs and app developers. For the latter, the questions were further divided to suit the HCWs and app developers. The first part contains their personal sociodemographic information. The second part contained specific open-ended questions on both HCWs’ and app developers’ perspectives regarding users’ awareness and understanding of mHealth apps. Meanwhile, HCWs were asked specific questions on their experience with the use of mHealth apps installed in patients’ personal devices, knowledge of patients’ health data security and privacy, benefits of mHealth apps to their patients, and potential issues that may prompt the patient to stop using the apps. App developers were probed on the security features that may increase the risk of data breaches and those that could be used to mitigate such risks. For the participating patients, similar questions were asked, except that the items were directed to them. The interview questions were presented in English and no translation to Arabic was performed.

Interviews were scheduled with the participants upon communicating with them via email or contact directories. Participants were provided with detailed information about the study and their rights, including the right to withdraw at any time without penalty. The interviews were conducted via videoconferencing platforms such as Zoom (Zoom Communications, Inc) or Google Meet, as preferred by the participants. Once the interview session commenced, participants were allowed to share their views on data privacy, confidentiality, and security issues relating to mHealth app usage. Interviews were audio-recorded, professionally transcribed, and checked for accuracy by another reviewer (NA). Data saturation was attained when no additional information was provided by the participant. It took approximately 25 minutes for each participant to complete the interview.

### Ethical Considerations

The ethical approval for this research was obtained from the Biomedical and Scientific Research Ethics Committee, University of Warwick (BSREC 03/22‐23) and the Medical Research & Ethics Committee, Ministry of Health in Saudi Arabia. The interview was designed to ensure that participants’ confidentiality was maintained throughout the research. Informed consent was obtained from all participants prior to the interviews. Videos were turned off during the interview, and only the audio was recorded. Thus, no identifying details were obtained from the participants during the research process. The researcher also stated the confidentiality of all information provided by the participants. Participants were informed that they could decide to withdraw from the study at any time without any penalty. No compensation or incentives were given to the participants. All collected data were stored on a personal computer with a secure password and were accessible only to authorized parties (the supervisory team and the researcher).

### Data Analysis

Data obtained from the interviews were analyzed using the NVivo software (version 12; QSR International)—a qualitative data analysis software used to organize, code, and identify themes from transcribed data. A thematic analysis was performed in which participants’ responses during the interview session were analyzed in-depth and checked for any interconnection. Some of the quotations were translated from Arabic to English because a few participants responded to some questions in Arabic despite being informed that the interview would be conducted in English. We also edited the quotations lightly for clarity while ensuring that the content is maintained. Transcription was performed by a research assistant who was not involved in the interview process.

An inductive approach was used to synthesize themes directly from the transcribed data by following the procedures described by Braun and Clarke [25], which is considered a robust systematic guide for thematic analysis. First, the data were scrutinized for familiarity, followed by coding systematically and synthesizing subthemes and themes. The generated themes were cross-checked with the participants’ verbatim statements, leading to the development of an initial thematic map. Next, the themes were refined and regrouped to address issues with some unsuitable codes and yield metathemes for more detailed grouping. Finally, the themes were defined and given a name. Thematic analysis was conducted by the first author (NA), and validation of the codes and themes was checked by the second author (MA). All other authors discussed the codes and themes, while discrepancies were addressed through consensus. All the experts and members of the research team discussed and agreed on the emerging themes and interrelated responses.

## Results

### Respondents’ Demographic Characteristics

This study involved 25 participants, comprising HCWs (n=11), mHealth app developers (n=2), and users (n=12). As shown in Table 1, male participants accounted for more than two-thirds of the samples, but they were evenly distributed in terms of age groups. All the participants were married, employed, and with at least a university or higher educational qualification. All the HCWs and app developers were active users of mHealth apps. Although 3 of the 12 patients or users were passive users, they were all recruited from hospitals where they are currently seeking medical assistance. However, despite considering nonusers of mHealth apps in this study, none of the recruited participants was identified as a nonuser. Apart from HCWs and app developers, the remaining participants were patients visiting the selected hospitals in which mHealth apps are primarily used for clinician-patient communication.

**Table 1.** Demographic characteristics of the respondents^a^.

Variables	HCWs^b^ and app developers	Patients/users
Sex		
Male	10 (76.9)	8 (66.7)
Female	3 (23.1)	4 (33.3)
Age (years)		
18‐30	5 (38.4)	4 (33.3)
31‐40	4 (30.7)	5 (41.7)
≥41 years	4 (30.7)	3 (25.0)
Marital status		
Single	0 (0.0)	0 (0.0)
Married	13 (100.0)	12 (100.0)
Educational qualification		
Primary/secondary	0 (0.0)	0 (0.0)
University/higher education	8 (61.5)	10 (83.3)
Postgraduate	5 (38.5)	2 (16.7)
Employment status		
Employed	13 (100.0)	12 (100.0)
Unemployed	0 (0.0)	0 (0.0)
Type of user		
Active	13 (100.0)	9 (75.0)
Passive	0 (0.0)	3 (25.0)
Nonuser	0 (0.0)	0 (0.0)
Currently a patient/HCW attending a hospital in Saudi Arabia		
Yes	11 (100.0)	12 (100.0)
No	0 (0.0)	0 (0.0)

a Values are presented as n (%).

b HCWs: health care workers.

### Thematic Findings

The main themes synthesized from the qualitative study are the following: (1) discordant perspectives on the understanding and awareness of mHealth apps, (2) perspectives and awareness of security and privacy of mHealth apps, (3) perceived benefits of mHealth apps, (4) challenges experienced when using mHealth apps (technical difficulties, data security, and data breach), and (5) suggestions to improve the security of mHealth apps. Textbox 1 shows the theme and respective subthemes.

Textbox 1. **Themes and subthemes synthesized from the qualitative analysis.**

Theme 1: Discordant perspectives on the understanding and awareness of mobile health (mHealth) apps

Theme 2: Perspectives and awareness of security and privacy of mHealth apps

Subthemes

- Positive perception
- Negative perception
- Health care providers’ responsibilities
- Poor knowledge and the need for patient or user education
- Collaboration with cybersecurity

Theme 3: Perceived benefits of mHealth apps

Subthemes

- Patient autonomy
- Remote monitoring
- Self-care management and medication adherence
- Time and cost-saving

Theme 4: Challenges experienced when using mHealth apps

Subthemes

- Data security and privacy issues
- Data breaches
- Technical difficulties

Theme 5: Suggestions to improve the security of mHealth apps

- Improved security features
- User-friendly features and tailoring toward patient needs
- Online consultation for emergencies
- Remote monitoring features

### Discordant Perspectives on the Understanding and Awareness of mHealth Apps

The first question assessed participants’ understanding and awareness of mHealth apps. Discordant perspectives were shared by respondents. Specifically, all HCWs and developers of mHealth apps demonstrated a good understanding and awareness, while patients demonstrated otherwise. Overall, HCWs depicted good mHealth app awareness and understanding while emphasizing that such a level of exposure was lacking among their patients. The main subthemes include factors influencing the usage of mHealth apps, poor awareness among users or patients, and the need for patient education. Some of the comments highlighting this theme are illustrated in the following quotations:

*I understand that there is low awareness of mHealth apps among patients. Patients have negative attitudes toward the use of mHealth applications because of various issues, including cost, security concerns, and uncertainty regarding which apps to choose. There is a need to promote mHealth app use among patients*.

[Health care professional, No. 13; Health care worker 13]

*My patient has been using a mobile health application for the last couple of years and is well acquainted with the usability of mhealth apps*.

[Health care worker 11]

Most users who reflected a good understanding and awareness of mHealth apps emphasized the role of health care providers in data collection, using the garnered data for remote health monitoring, the positive impact of COVID-19 disease on increased usage of telehealth, and general information on the app operations and functionalities.

*These apps can provide a range of services, including helping users track their physical activity and nutrition, providing educational resources on various health topics, and offering tools to manage conditions such as diabetes or mental health*.

[mHealth app user, No. 10: US 10]

On the other hand, a few users opined that they were unsure of the reason and purpose for collecting their data. Examples of the responses reflecting users’ poor understanding and awareness of mHealth apps are presented as follows:

*I do not have a deep understanding of mobile health applications, but little that I know is that Mobile health (mHealth) applications are software programs that run on smartphones, tablets, and other mobile devices and are designed to support health and healthcare-related functions*.

[US 7]

### Perspectives and Awareness of Security and Privacy of mHealth Apps

#### Overview

Five subthemes were synthesized from the users’ responses regarding their perspectives and awareness of mHealth apps’ data security and privacy. These subthemes included collaboration with cybersecurity organizations, positive perception, negative perception, health care providers’ responsibilities, and poor knowledge of data security and privacy.

#### Positive Perception

Six of the 10 users perceived that the security and privacy of their data are strictly protected by health care providers and used for the purpose they were collected. These respondents also posited that the database is secure and prevents unauthorized or third-party access to their data. Furthermore, users cited examples of the security features that prompted their positive feedback on mHealth apps, as well as relaying the actions taken by health care providers to ensure the confidentiality and security of patient data.

*I believe to a large extent my data are secured and well-protected because I take my time to navigate through the apps and see the security features available in the app before I start using it*.

[US 9]

*The security features strongly protect users’ data because the app developers provide a range of data-protective mechanisms. Moreover, the data requested is basic regarding my health condition*.

[US 2]

#### Negative Perception

On the other hand, 4 of the 10 users opined that the security and privacy of patient data were far from being protected, thus increasing the risk of data breaches and unauthorized access. Among the issues raised by respondents are potential usage of data for nonmedical purposes, ambiguous privacy policies that make it difficult for users to understand how data privacy and security are handled, and a lack of sensitization by health care providers. Therefore, this group of respondents recommended that developers of mHealth apps and health care providers should collaborate to ensure enhanced protection of devices.

*I am afraid of the usage of my data for other things besides the medical and communication with healthcare professionals. This is a critical time whereby data can easily leak to hackers*.

[US 6]

*It’s a two-way event because data can be used to improve the delivery of healthcare services, but at the same time, unauthorised access to such data may have serious implications, especially if manipulated and used for other purposes*.

[US 10]

#### Health Care Providers’ Responsibility

This theme was synthesized mainly from HCWs’ responses to the second interview question. Five of the health care professionals highlighted that it is their responsibility to enlighten patients on the available security features on their mHealth apps. They also claimed to be responsible for any data breach since they are considered the custodians of patient data.

Health care professionals also raised concerns regarding data security and confidentiality, especially challenges in managing patient data. They highlighted the limitations of the current security features in mHealth apps, which may heighten the risk of data breaches and violation of patient privacy.

*It is our duty to ensure that patients’ data are protected and used solely for medical purposes. Most of these patients are naïve and not really exposed to mHealth apps because of their backgrounds. Hence, we have to educate them when we have the opportunity to do so*.

[Health care worker 5]

*Although the app developers are responsible for designing the security and data privacy features, I believe our role as clinicians is important to mitigate any practices that may lead to data confidentiality and privacy issues. The level of access to patient clinical data needs to be moderated across levels of healthcare services*.

[Health care worker 2]

#### Poor Knowledge and the Need for Patient Education

While some users demonstrated either positive or negative attitudes toward data security and privacy, a few participants exhibited poor knowledge of these issues. This theme was reflected in users’ lack of understanding of the security features available in their devices and their role in ensuring that their data are not shared with unauthorized parties. This finding is reflected in the following statements:

*What is more important to me is to manage my health condition. I don’t think I deeply understand the provisions in the apps to address the possibility of my personal data falling into the hands of authorised persons. Seriously, I want to learn more because it’s the primary duty of the clinicians and app designers to protect my data, right?*

[US 8]

*I can’t even recall any information relating to the privacy and security of these apps, despite being a user for almost 2 years. I just focus on using it to book appointments and update my treatment progress*.

[US 4]

As a result, health care professionals and mHealth app developers emphasized the need for more patient education. Accordingly, such interventions should be tailored toward patient needs upon identifying their shortcomings in data security and privacy. This includes proper usage of security features, strong passwords, and who they can share their data with to prevent data breaches.

*We have to educate patients on how to navigate through the security features and use strong passwords to protect against unauthorised data access. This is more important at the moment because there are several websites and apps other than mHealth apps requesting patient personal data*.

[Health care worker 5]

#### Collaboration With Cybersecurity Organizations

The fifth theme uncovers the need for health care providers to collaborate with cybersecurity institutions to improve the security, privacy, and confidentiality of data collected via mHealth apps. These respondents did not relay either a positive or negative perspective toward data security and privacy but mainly recommended the approach that can be adopted by health care providers to address the issue. A similar position was shared by the stakeholders who advocated for networking and collaboration between various health care bodies to enhance data security and privacy. The following statements reflect the emerging theme:

*I strongly recommend working together with mHealth app developers, given our experience and understanding of the issues raised by patients and users of these apps. We are in the best position to share the information with the developers since we communicate frequently with patients*.

[Health care worker 8]

*It will be challenging for mHealth app developers to enhance the security features of the digital technology without our input as clinicians from the point of patient-clinician relationships. Patients don’t have access to the developers of these apps; hence, they often share their concerns with other users or healthcare workers during consultation*.

[Health care worker 1]

### Challenges Experienced When Using mHealth Apps (Technical Difficulties, Data Security, and Data Breach)

Pertinent information was gleaned regarding the challenges faced by health care professionals and patients while using mHealth apps. These barriers ranged from data security and privacy issues to data breaches and technical difficulties. Poor usability encompasses problems with navigation, understanding the features, and slow operation. These problems made some patients prefer visiting their doctors since the app was not effective in delivering mHealth services. These findings are captured in the following statement:

*I had some challenges navigating through some features. Sometimes I preferred visiting the doctor since I needed to utilise the application to suit my needs effectively. When using the application, I was unsure if my data records were safe since there have been increased hacking and theft cases that could easily compromise my medical records. On rare occasions, the application could freeze, and I needed more time to get what I needed*.

[US7]

A similar scenario was highlighted by health care professionals, who claimed that the app could be misleading with usability, especially when notifications are delayed and during data updates. These challenges are also linked to the issues of violation of data privacy, incidents of data breach, and technical difficulties.

*The mHealth app can sometimes be misleading, especially when the notifications are delayed. I have also experienced misfunctions of the app when updating data and printing some information. I have heard instances where the app stopped operating, asking for updates, which could lead to catastrophe in the case of a terminally ill patient*.

[Health care worker 2]

*I have been approached by many patients who wanted me to teach them how to use the application since they did not know how it functions. Even after teaching some of them how to use the application, I experience other issues, such as security and privacy and inaccurate results that patients experience. Some of the applications did not function well, and they frustrated the users*.

[Health care worker 12]

### Perceived Benefits of mHealth Apps

#### Overview

It is well established that mHealth apps offer several advantages and benefits, especially during and in the post–COVID-19 era. Four subthemes were identified, namely, patient autonomy, remote monitoring, self-care management and medical adherence, and time and cost-saving. These themes encompass both HCWs’ and patients’ responses to the interview questions. The following subsection discusses each theme.

#### Patient Autonomy

The term “patient autonomy” was used by 3 HCWs in this study while describing the personalization of health care access and services for patients. This theme also elucidates patient control over their data. Likewise, 1 user agreed that mHealth apps have enabled them to make personal changes to their medical data, which can be detected only by the health care provider.

*Patients have a higher degree of autonomy. The applications have also helped many patients to save money and time while quickly accessing medical help*.

[Health care worker 5]

*Mobile health applications are an essential tool that has enabled me to personalise my healthcare access. It is a time-saving tool that helps me quickly get the necessary healthcare services. I can easily book appointments and manage my prescriptions and other healthcare services*.

[US 6]

*There is a tendency for patients to perceive that their data are secure once they experience the benefits of mHealth apps in terms of treatment and management of their health conditions. This is what I have noticed over time since we introduced these apps to clients*.

[Health care worker 4]

#### Self-Care Management and Medication Adherence

This theme was among the most recurring information provided by health care professionals (n=6) and users (n=5) of mHealth apps in this study. Apart from improving patient awareness of the functions and importance of mHealth apps, several users relayed that the technology has assisted them in adhering to various clinicians’ recommendations and disease management modalities. This includes adhering to recommended diet, medication, and lifestyle activities, therefore culminating in self-care management. Most importantly, the users with this view posited that their health has improved significantly due to the self-care management features provided on the app. The following statements reflect the present theme:

*The mHealth app has proven to be an invaluable tool in my journey towards better health. It has allowed me to adhere to a strict diet, keep track of doctors’ appointments and medication schedules, and monitor my metabolism and heart rate*.

[US 7]

*The Mhealth application provided me with timely medication, which led to the improvement of my health condition. I was able to attend to my health using the application easily*.

[US 9]

#### Remote Monitoring

The fact that mHealth apps provide the opportunity for patients and health care professionals to communicate without physical visits was mentioned as a key benefit by both parties. Stakeholders reported that this benefit contributed to the increasing trend of mHealth adoption and usage following the COVID-19 pandemic. Thus, clinicians were able to monitor and track their patients irrespective of their locations. Patients also benefit from communicating directly with clinicians without hospital visits.

*The app has helped me to stick to certain routines, allowing efficiency. The app has also helped me to monitor patients, such as those at home, without having to visit them. The app has also allowed me to keep my record by downloading history easily without having to ask the patient*.

[Health care worker 3]

*The mHealth app has helped me to monitor the progress of the patient without having to be there. The app allows sending suggestions and prescriptions, and the recording of data more dynamically and easily*.

[Health care worker 2]

*The application kept my health in check while reducing my re-visits. I also saved time costs related to physical visits to the hospital*.

[US 4]

#### Time-Saving and Cost-Saving

In line with the provisions for remote monitoring and self-care management, mHealth apps offered a crucial benefit by facilitating real-time information access without any physical interaction between the health care professional and patients. These events save both parties from expending time and resources associated with hospital visits. According to health care professionals, this provision of mHealth apps is crucial in the management of patients with chronic diseases and those requiring home-based therapy.

*The application kept my health in check while reducing my re-visits. I also saved time costs related to physical visits to the hospital*.

[US 4]

*The app has also provided a convenient way for me to track my progress, and my doctor has been able to monitor my health remotely, saving me both time and money*.

[US 9]

### Suggestions to Improve the Security of mHealth Apps

#### Overview

Based on the diverse issues raised by users and stakeholders regarding the challenges faced in using mHealth apps, the respondents were probed on the features that need to be integrated into these advanced health technologies to address the identified issues. Resultantly, 4 subthemes emerged from the thematic analysis as follows: improved security features; user-friendly features and tailoring toward patient needs; online consultation for emergencies, tailoring toward patient needs; and remote monitoring features. Each of these themes is presented in the following section.

#### Improved Security Features

Several participants (HCWs: n=4, users: n=5, and App developers: n=1) suggested the need to add more security features to enhance the privacy, security, and confidentiality of data collected via mHealth apps. Different security options and features were mentioned by respondents, including IP address, notification, and second-step verification such as OTP, thumbprint, or facial recognition. Some respondents stated that the app notification system should be enhanced such that the notification is received by the doctor and the patient. These positions were held by patients and health care professionals as presented in the following comments.

*The greatest threat to these apps is security and privacy. To ensure that this is checked, the app should have a change of IP address notification whenever a new device is used, seeking verification. The app should also have a second-step verification whenever accessing the app, such as a thumbprint or OTP*.

[US 6]

*Let’s improve the security of operating these apps by allowing thumbprint or facial recognition as a form of password to manage these apps. The apps should also come with a printing option so the patient can print, for example, a week’s data on their progress*.

[Health care worker 5]

*We are currently working on how notifications can be sent to the healthcare provider and patient, as a way of enhancing the security system and data shared between both parties*.

[App developer 2]

#### User-Friendly Features and Tailoring Toward Patient Needs

Another important theme emerging from the analysis is to make the mHealth app user-friendly. Participants posited that user-friendly features may redirect patients’ focus to the usability of the mHealth apps rather than focusing on security and privacy issues. This suggestion was presented by 3 users and 4 HCWs who emphasized the features to encourage the usability of the app and shape the users’ attitudes and perceptions toward the security and privacy of such apps. Examples of the features include font size, navigation icons, language preference, and login procedures. A few verbatim statements reflecting this theme are shown in the following quotations:

*The features I expect to be added include making the app user-friendly. This involves personalising the app depending on the patient’s characteristics, such as age, language preference, and technical literacy. This can help advance the usability of the app*.

[Health care worker 4]

*A user-friendly interface should be incorporated into the mobile health application. This would help ensure that the system is easy to use*.

[US 2]

This subtheme also reflects the need to consider patient needs when introducing any new feature to the current mHealth apps used in Saudi Arabia. This position was mostly held by HCWs as they suggested tailoring the app to suit users’ age, language preference, and technical expertise. Home-based health care services were also suggested to be integrated into mHealth apps to accommodate patient needs. In addition, 1 app developer recommended designing specific features to encourage self-care management among patients with chronic diseases. Respondents claimed that these features will not only improve users’ interactivity and usability but also provide a medium for social support and increase patient control of their data.

*Patients with chronic illness can have their basic pulse and BP readings monitored after integration with their Fitbit app. This feature will allow them to quickly get the assistance they need at any place and at any time. The patients can read their pulse during their regular duties and check their blood pressure*.

[App Developer 1]

#### Online Consultation for Emergencies

Three users and 2 HCWs advocated for a feature specifically designed to enable online consultation for emergency patients. This suggestion stemmed from the challenges associated with the usability of the apps, which may be time-consuming and non–user-friendly. The corresponding comments are presented in the following quotations:

*An online consultation will be only for emergencies for our registered patients*.

[US 1]

*I believe a function in the app should be dedicated to emergency cases, to enable faster response*.

[Health care worker 5]

#### Remote Monitoring Features

Respondents (n=4) opined that more features are required to ensure effective remote monitoring via mHealth apps. Some respondents felt that the features are limited probably to reduce the risk of data security and privacy issues. This theme also emerged from respondents who had negative perceptions of current mHealth apps used in the country. Apart from improving data security and preventing data breaches, this group of respondents suggested better features to monitor both admitted patients and outpatients, as well as motivating patients to engage in self-care management.

*Maybe it is because of the concerns regarding the security of these apps, but I think it does not really help for online communication with healthcare providers. The apps need to be improved for real-time tracking of outpatients and follow-up without exposing our data to unauthorised channels*.

[US 3]

*I am using the NahdiCare apps, which are mainly for monitoring care. There is no provision for detailed interaction with the clinician. Most times, I have to visit the hospital for issues that should be resolved via the app*.

[US 10]

## Discussion

### Principal Findings

This study constitutes the first attempt to explore users’ perspectives on issues relating to the privacy and security of data collected via mHealth apps in Saudi Arabia. Besides, there is limited information on how these challenges may affect the adoption and utilization of digital health technologies. In this study, we focused on the users and nonusers of mHealth apps, as well as HCWs and app developers who constitute the predominant stakeholders of these advanced digital technologies in Saudi Arabia.

### Awareness and Understanding of mHealth Apps

Regarding the awareness and understanding of mHealth apps, stakeholders in the health care sector, such as HCWs and app developers, demonstrated a good understanding of issues relating to data security and privacy, whereas users and patients were perceived to lack such knowledge. Moreover, patients also demonstrated a diverse understanding of mHealth apps, which aligns with several studies reporting users’ concerns about the security and privacy of sensitive data, including images of body parts and those revealing their identity [22,24,26]. A systematic review by Aljohani and Chandran [13] reported that the ease of access and operation of mHealth apps were among the reasons for hospitalized patients’ concerns about unauthorized access to their health data [13]. However, some patients were not worried about these issues either because of their confidence in data safety and features to prevent data breaches, or they were completely unaware of the potential risks involved. This constitutes an important finding, given that most users in this study were highly educated, indicating possible disturbing levels of ignorance and awareness among medium to noneducated users in the general public. Users who are unaware of data privacy and security features and provisions in mHealth apps may be unable to differentiate between risky behaviors, depending on the type of private data collected by various technologies [27].

On the contrary, the understanding and awareness shown by HCWs are expected since they are more conversant with mHealth apps. They are also more exposed and educated, thereby increasing their perception of the risks and benefits associated with digital health technologies. In order to address the low awareness level among patients, most HCWs suggested the need for patient education and enlightening them on pertinent areas. Numerous studies have taken the same stand by emphasizing brief training or education on data security and assuring data confidentiality and privacy [27-29]. Both Nurgalieva et al [30] and Lewis and Wyatt [31] recommended that patients should be trained and educated to address the external risk factors associated with the violation of users’ privacy and security in mHealth apps.

### Recommendations to Improve mHealth Apps’ Data Privacy and Security

All the groups of participants in this study provided insights into measures to improve the privacy and security of data used in mHealth apps. Examples include features to improve data security and remote monitoring, making the app more user-friendly and tailored toward the patients’ needs, and providing online consultation for emergencies. Specific security features such as 2-step verification and authentication, as well as the use of thumbprints and strong password requirements, were mentioned by health care professionals and end users. In the same vein, Alwashmi et al [32] opined for the use of an alphanumeric passcode to ensure the protection of mHealth apps rather than using a 4-digit pin, as well as wiping data from the mobile device after a specific number of failed passcode attempts. Nurgalieva et al [30] also emphasized the frequency of notifications and alerts programmed into mHealth apps.

As observed in this study, participants recommended sending notifications to both the health care provider and the patient to ensure extensive review of login attempts or data sharing. A discreet or private notification was advised to prevent any distress to users, particularly in situations where someone mistakenly views the icon. Overall, these recommendations reecho the need for users to have complete control in using their smartphone for mHealth and avoid any intrusion into their daily lives. This event coincides with the personalization and tailoring of newly added features according to patient needs, as mentioned by some respondents. Certain adjustments to the security and privacy features of mHealth apps need to be incorporated by the app developers upon considering patients’ and users’ demographics, as evident in previous studies [4]. However, given the low number of studies reporting the underlying factors influencing patients’ views on data privacy, confidentiality, and security issues in mHealth apps, more research is needed to elucidate the relationships.

Another key recommendation, especially by users, was the need for real-time online consultations for emergency cases. This suggestion stems from the challenges faced in navigating through mHealth apps, which may be time-consuming and ineffective for health care service delivery. Therefore, a specific platform on mHealth apps to facilitate easy and prompt communication with the health care provider’s emergency unit was advocated. Such an intervention will come with several benefits and challenges, particularly in terms of educating users. Regarding the benefits and experience garnered from using mHealth apps, participants mentioned diverse ways in which they have benefited from mHealth apps. It is well established that mHealth apps offer several advantages and benefits, especially during and in the post–COVID-19 era. The subthemes included patient autonomy, remote monitoring, self-care management and medical adherence, and time and cost-saving.

Patient autonomy refers to equipping patients or users, as the case may be, with substantial control of their data. Several users and health care professionals highlighted this view either by using personalization of patient data or by giving them unlimited access. On the other hand, remote monitoring entails online interaction and examination between patients and health care professionals. Consequently, patients were motivated to engage in self-care management and medication adherence, ranging from assessing physical parameters to restricting diet. These events contributed significantly to efficient disease progress monitoring, good communication between patients and health care professionals, and better patient outcomes. Our results corroborate the reports from previous studies, whereby improved health status [33] and better patient-health care professional relationships [34,35] were stated as the main benefits of using mHealth apps.

In terms of participants’ experiences with mHealth, although encouraging comments were highlighted by several users, important barriers were mentioned that require further discussion. These barriers ranged from poor usability to data security and privacy issues, data breaches, and technical difficulties. Concerns about data privacy and confidentiality may discourage patients and end users from using mHealth apps [35]. Likewise, the low levels of privacy and security of patient data contributed significantly to the low adoption rate among the intended users [13]. Past reviews also reported security incidents associated with mHealth apps, such as vulnerabilities to data breaches and malware attacks. The concerns raised by the participants are plausible based on the extensive usage of mobile devices with location sensors in Saudi Arabia, which has facilitated easy access to location-based services [33]. Given the extensive privacy and security issues raised by the stakeholders in this study, cyberspace security can be further explored to improve the security of mHealth environments [4].

Surprisingly, the challenges faced appeared not to deter participants from using the technology, as all the respondents who shared their concerns were active users of mHealth apps. A recent study conducted among mature adults also shared a similar view, as none of the participants stopped using the technology despite their data privacy concerns [27]. This aligns with the widely discussed privacy paradox [36], whereby people engage with technology even when they acknowledge its potential privacy loss issues. Research involving social media, e-commerce, and mobile apps has also demonstrated this phenomenon [37,38]. This paradoxical behavior is likely driven by users’ perception that the immediate benefits of using mHealth apps outweigh their potential or hypothetical risks [27].

### Strengths and Limitations

This study represents the first attempt to qualitatively explore patients’ and stakeholders’ perspectives regarding the privacy and security of data collected via mHealth apps in Saudi Arabia. Our findings offer insights on possible strategies to improve end users’ and patients’ adoption of mHealth apps and addressing concerns relating to data privacy and security. In terms of sampling, purposive sampling was used to recruit participants with diverse backgrounds (age, gender, app experience, and multiple hospitals). This approach aligns with qualitative research goals of capturing variation and depth. Moreover, qualitative studies are not designed to achieve representativeness or collect data from a representative sample.

Nevertheless, this study has several limitations that need to be acknowledged and considered when extrapolating the findings. We developed the interview guide and conducted the interviews in English, and only a slight translation was performed during the data transcription. Based on this criterion, most of the respondents were highly educated. As for the HCWs and app developers, this procedure is unlikely to introduce a selection bias since these professionals are expected to hold a higher educational level. However, most users and patients also had at least a university degree, indicating that their views on mHealth and privacy may not be representative of the general Saudi population. Nevertheless, it is interesting to note that several users were still unaware and had a limited understanding of privacy and security of mHealth apps—possibly reflecting a more serious problem in the general population.

Despite our plan to include nonusers in the study, all the participants were either active or passive users of mHealth apps. The patients recruited were from hospitals in which mHealth apps are actively used for consultation and follow-up, making it difficult to identify those who are yet to adopt these digital health apps. Therefore, our findings present limited perspectives on barriers among nonusers. This limitation narrows the study scope and insights. Future studies may consider recruiting nonusers of mHealth apps from the Saudi community or hospitals where patients are not compelled to use such apps.

Our interview guide was limited in terms of exploring security issues, which led to the superficial technical aspects raised by the app developers and HCWs. While patients were less aware of these technical aspects, as evidenced from the interviews, app developers and HCWs occasionally mentioned the need for more comprehensive access controls, whereas other critical aspects, such as data storage, encryption, and server-side protection, did not emerge from their responses. Apart from the limited questions that may trigger deeper insights into security-related issues, only 2 app developers were recruited, and HCWs may have limited technical awareness while focusing on health-related data and care delivery when using mHealth apps. Future studies may bridge this research gap by recruiting a larger number of stakeholders comprising HCWs and app developers who are technologically savvy for a nuanced understanding of technical and security issues associated with these digital health technologies.

### Research and Practical Implications

The results from this study have important implications for mHealth app stakeholders in Saudi Arabia. Both HCWs and developers of mHealth apps have vital roles in addressing the challenges identified in this study, particularly issues relating to data privacy, confidentiality, and security that may affect the adoption and usage of digital health technologies.

Since HCWs acknowledge patients’ low awareness and understanding of mHealth apps and the data collected in the process, they can assist by educating patients to address such knowledge deficits. Patients could be briefed during routine consultations by highlighting the need for recording sensitive data and the purpose of collecting such information. Regarding unauthorized third-party access and potential data breaches, users may benefit from information on the privacy requirements and meeting the standards set by the GDPR and HIPAA, which are designed to ensure that such privacy issues and data breaches are prevented.

Another important aspect is training and educating end users on the existing features and measures to ensure that their data remain secure and confidential. Nevertheless, educational interventions need to be effectively tailored toward the user’s level of awareness and understanding. As evident from this study, users’ less concern about their data privacy and security may arise from being completely ignorant of these potential challenges, or they may perceive that the benefits of using mHealth outweigh the potential risks.

mHealth app developers can also benefit from the research findings, as the concerns raised by patients and end users may culminate in low adoption and usage of mHealth apps [18]. While users are expected to express concerns regarding data privacy and security, it remains unclear whether such concerns influence their behavior and actual use of such apps. This gray area needs more understanding and research. mHealth app developers, particularly in Saudi Arabia, have the opportunity to digest the issues raised by users and strategize on how to improve their current security and privacy measures. Such strategies should be designed and implemented to meet the requirements of HIPAA.

Finally, policymakers can use the research findings in identifying the areas to focus on when developing policies and strategies to facilitate the wide adoption of mHealth apps and achieve the desired outcomes. Accordingly, policymakers can emphasize features to enhance better patient–health care professional relationships, trust, and personal data protection and ensure that health status or medical conditions are not disclosed, as well as brief training or education on data security. Although these points can be relayed to users, trust is a feature that has to be earned, which has to be driven by HCWs and the government.

## Abbreviations

CIS: clinical information system
GDPR: General Data Protection Regulation
HCW: health care worker
HIPAA: Health Insurance Portability and Accountability Act
mHealth: mobile health
